# Office-Cycling: A Promising Way to Raise Pain Thresholds and Increase Metabolism with Minimal Compromising of Work Performance

**DOI:** 10.1155/2018/5427201

**Published:** 2018-01-23

**Authors:** Rebecca Tronarp, André Nyberg, Mattias Hedlund, Charlotte K. Häger, Suzanne McDonough, Martin Björklund

**Affiliations:** ^1^Department of Community Medicine and Rehabilitation, Physiotherapy, Umeå University, Umeå, Sweden; ^2^Institute of Nursing and Health Research, Ulster University, Jordanstown, UK; ^3^Centre for Musculoskeletal Research, University of Gävle, Gävle, Sweden

## Abstract

**Aim:**

Establishing the effects of low intensity cycling (LC), moderate intensity cycling (MC), and standing at a simulated office workstation on pain modulation, work performance, and metabolic expenditure.

**Methods:**

36 healthy adults (21 females), mean age 26.8 (SD 7.6) years, partook in this randomized 3 × 3 crossover trial with 75 minutes of LC on 20% of maximum aerobic power (MAP) output, 30 minutes of MC on 50% of MAP, and standing 30 minutes with 48-hour wash-out periods. Outcome measures were pain modulation (pressure pain threshold (PPT) and thermal pain threshold)), work performance (transcription, mouse pointing, and cognitive performance), and metabolic expenditure.

**Results:**

PPTs increased in all conditions. PPT trapezius showed the highest increase after LC, 39.3 kilopascals (kPa) (15.6; 78.6), compared to MC, 17.0 kPa (2.8; 49.9), and standing, 16.8 kPa (−5.6; 39.4), *p* = 0.015. Transcription was reduced during LC and MC. Mouse pointing precision was best during standing and worst and slowest during MC. Cognitive performance did not differ between conditions. Metabolic expenditure rates were 1.4 (1.3; 1.7), 3.3 (2.3; 3.7), and 7.5 (5.8; 8.7) kcal/minute during standing, LC, and MC, respectively (*p* < 0.001).

**Conclusions:**

LC seems to be the preferred option; it raised PPTs, more than doubled metabolic expenditure, whilst minimally influencing work performance.

## 1. Background

The negative health effects of a sedentary lifestyle have been increasingly highlighted in the last decade. According to the World Health Organisation (WHO), physical inactivity is nowadays considered the fourth leading risk factor for global mortality [1] and detrimental associations have been observed between sedentary time and several lifestyle related diseases [2–6] and all-cause mortality [7]. Alarmingly, the negative health effects do not seem to be compensated for by shorter bouts of high intensity exercise [8]. Indeed, recent evidence suggests that sedentary individuals can attenuate the risk by engaging in at least 60–75 minutes of moderate activity daily [9], which is likely challenging for many persons. Low physical activity levels and prolonged sedentary work time/hours have also been emphasized as a risk factor for developing musculoskeletal (MS) pain [10, 11], more specifically of the neck and shoulder [12]. In Sweden, MS disorders are the most prevalent cause for long term sick leave amongst men and the second most prevalent cause amongst women [13]. MS disorders are also a predominant issue within European society [14] explaining roughly 38% of the costs of work-related absenteeism [15]. Further, the majority of people with upper extremity disorders report a productivity loss with an average reduction of 34% [16]. The fact that occupations in general are becoming more and more sedentary [17], which causes negative health effects along with detrimental associations with MS pain, makes the workplace an important arena for multipreventive interventions [18], for example, in the form of the so-called active workstations.

Usage of treadmills, at present the most commonly researched type of active workstation, has been shown to lead to reductions in sedentary time [19, 20], increased low intensity physical activity, and thus increased energy expenditure during the workday [21–28], as well as positive effects on different health parameters [19, 20, 23]. However, several studies have also demonstrated negative effects on the users work performance [29–31], and the need for alternatives has been highlighted [31]. A few studies have examined other types of active workstations and shown that cycling workstations seem to be a feasible option [32] as a potential way of reducing sedentary time whilst instead increasing the time of low intensity activity during the workday [33]. To date only a few studies have investigated the effects on work performance whilst using a cycling workstation [31, 34–36] showing inconclusive results. Typing performance has either been unaffected [34, 36], improved [35], or slightly compromised [31] during cycling when compared to sitting. Mouse pointing has been shown to be slightly compromised during cycling when compared to sitting [31]. Cognitive performance on the other hand has either been unchanged [35] or improved [36] during cycling when compared to sitting.

Aerobic exercise has shown altering pain modulation systems of the body, inducing a phenomenon called exercise-induced hypoalgesia (EIH). EIH has been reported in healthy persons [37–39], in physically active and inactive persons [40], and in individuals with chronic MS pain [41]. In the absence of direct evidence a narrative review has emphasized the strong theoretical rationale for exercise to be used in treatment of central sensitization of pain [42]. Such assumptions lead to still unanswered questions of which dosage, intensity, and time periods would be necessary. The shortest reported bout of aerobic exercise resulting in EIH is 15 minutes of cycling at 75% of VO_2_max [40] and analgesic effects have also been seen after cycling at 50% of the heart rate reserve during 25 minutes [39]. There is, however, a lack of knowledge regarding the effects of low intensity aerobic exercise with a longer duration, which probably would be a more suitable setup for active workstations. Also, there is only limited knowledge in the literature exploring whether exercise intensity at the chosen workstation influences work performance and thus has negative effects on work productivity.

Thus, the aim of this study was to establish, using our custom designed office-cycle work station in healthy men and women, the effects of low intensity cycling and moderate intensity cycling in comparison to standing, primarily regarding pain modulation and secondarily regarding work performance and metabolic expenditure.

We hypothesised that moderate and low intensity cycling would lead to increased pressure pain thresholds and that low intensity cycling would have less negative impact on work performance compared to moderate intensity cycling.

## 2. Method

### 2.1. Participants and Eligibility Criteria

Participants in this randomized repeated measures 3 × 3 crossover study were recruited through advertisement on bulletin boards at the campus of Umeå University, Sweden. Block randomization together with orthogonal Latin square for three treatments [43] was used to randomize and balance the study. The inclusion criteria were healthy individuals without any known cardiovascular, metabolic, neurologic, or lung diseases and without musculoskeletal or other pain, being >18 years old, and being accustomed to computer work. Exclusion criteria were intake of any medication affecting the heart rate and/or any pain medication. Eligibility was confirmed with the participants verbally or through e-correspondence. The participants were asked to refrain from tobacco and caffeine during the day of testing. The study was conducted according to the Declaration of Helsinki; all participants signed an informed written consent form prior to participating. All data was treated with confidentiality. Ethical approval was granted through the Ethical Review Board, Umeå, Sweden (2015/465-31).

### 2.2. Procedure

The study consisted of four sessions with standardized test protocols conducted in a laboratory with a simulated office environment. During the first session all participants performed an aerobic submaximal exercise test according the Åstrand-Rhyming method [44]. The other three sessions were experimental test conditions: standing (control condition), low intensity cycling (LC): cycling for 75 minutes at 20% of maximum aerobic power (MAP) output, and moderate intensity cycling (MC): cycling for 30 minutes at 50% of MAP at a height-adjustable office desk. To determine the duration of the low intensity cycling the estimated energy expenditure during a 30-minute bout of exercise on 50% of MAP was used to calculate the duration needed for the light intensity to result in a corresponding estimated metabolic expenditure. The experimental conditions were performed in a randomized order on different days separated by a wash-out period of 48 hours [39, 45].

#### 2.2.1. Submaximal Exercise Test

The workload for the test was individualized based on American College of Sport Medicine's recommendations for low-fit women and men [46] which resulted in workloads ranging between 50–125 and 75–150 watts for women and men, respectively. The participant cycled at a constant pedal cadence (50 revolutions*∗*min^−1^) for 6 minutes and rated their perceived exertion according to the Borgs ratings of perceived exertion (RPE) scale (rating interval 6–20) [47] at the completion of each minute of the test. The submaximal test and the two cycling conditions were performed using a custom-built office-cycle (OfficeBike™, a modified version of an ergometer cycle from Monark Exercise AB, Sweden, without handlebars, stable but with transporting wheels and thus designed to fit in an office environment). Heart rate was monitored continuously using a heart rate monitor band (Polar Electro Inc., Finland), which wirelessly connected to the office-cycle and logged the heart rate. The estimated maximum oxygen uptake (VO_2_max) was then used to mathematically derive MAP, that is, the power output (watt) corresponding to the estimated value of VO_2_max (l/min), by interpolation of the quadratic equation based on Åstrand-Rhyming data: −1.61*x*^2^ + 83.72*x* − 23.48, where *x* represents the estimated maximum oxygen uptake [48]. Finally, the participants performed one trial run of the three different computerized work performance tests used in this study as well as the pressure pain threshold test.

#### 2.2.2. Experimental Conditions

The full test procedure is described in Table 1. Pain modulation was tested on three occasions during each session: immediately before and after the activity [49, 50] as well as 30 min after finishing the activity [45]. During LC, the participants were asked to pause the pedalling after 30 minutes and pressure pain threshold (PPT) measures for trapezius were tested whilst the participant remained seated on the office-cycle; as soon as the PPT measures were taken the participant continued pedalling again. The participants carried out all work performance tests at 10 and 20 minutes following the start of the activity during standing and MC and in the LC condition the tests were repeated a third time after 65 minutes of cycling. This last LC test of work performance was 10 minutes before the end of cycling and corresponded to the last test occasion (at 20 minutes of activity) in both MC and standing. Measures of metabolic expenditure were registered continuously during the activities and rating of perceived exertion on the RPE scale was collected at set times during the activity (see Table 1).

### 2.3. Outcome Measures

#### 2.3.1. Primary Outcome Measure

*Pain Modulation—Pressure Pain Thresholds. *The primary outcome measure was change in pressure pain threshold (PPT) measured in kilopascal (kPa) using a Somedic Algometer (Somedic Productions AB, Sweden) with a 1 cm probe [51, 52]. The PPT measures were taken on three test sites: the right quadriceps femoris muscle (QF) 20 cm proximal to the patellar base [40, 41], the right ventral forearm (VF) eight cm distal to the cubital fossa [39], and the right upper trapezius muscle (TP) [41, 53] in the middle between the spinous process of C7 and acromion. The first two test sites were tested with the participant in a supine position and TP was taken with the participant in an upright sitting position. Pressure was manually applied perpendicularly using the handheld algometer with a continuous pressure increase of 40 kPa/s until the participant reached his/her pressure pain threshold [54], defined as the point when the pressure starts causing discomfort or pain [55]. Before pressure was applied with the algometer, the test leader palpated the test points bilaterally and if any unilateral tenderness was present on the right side the test point was moved according to a predefined schedule. Measures were taken three times at each site during all test occasions and a mean value of the two last tests was calculated and used in the statistical analysis [49, 56].

#### 2.3.2. Secondary Outcome Measures

*Pain Modulation—Thermal Pain Thresholds.* Thermal pain threshold (TPT) [57] was measured in degrees Celsius (°C) using a Thermotest® (v. 01-S, Somedic AB, Hörby, Sweden) with the participant in an upright sitting position. The thermode was placed at the two test points for TPT: the neck [58] and the thenar eminence of the nondominant hand [59]. The thermode had a starting temperature of 32°C; the temperature then either decreased or increased at a rate of 1°C per second depending on whether measuring cold pain threshold (CPT) or heat pain threshold (HPT) and was reset to starting temperature between each test. The participants were asked to press down a handheld switch as soon as the sensation first became painful, which then immediately ended the test. Due to safety reasons, the lower temperature limit was 5°C and upper limit 52°C. Each test point was tested twice for CPT and HPT and the mean value of the two tests was used in the analysis [59]. 

*Work Performance*. Typing performance was measured using a timed text transcription task, transcribing as much text with as few errors as possible during a two-minute period. The task was performed using a split-screen display typing software (Typing Master 10, TypingMaster Finland, Inc.) [31]. The results were presented as net (excluding erased typing errors) and gross (including erased typing errors) typing speed (words per minute (words/min)) as well as the number of typing errors.

Computer mouse pointing performance was estimated as the number of successful tasks and time to complete all trials. It was registered using an omnidirectional tunnel steering task (UW Pointing Device Testing Program, Version 1.9). Each test consisted of 10 trials and the task was to move the pointing device and click on a grey circle (4 mm radius), release the click, and move the circle as fast as possible through a 15 mm wide and 90 mm long tunnel, without touching the walls, and then leave the circle in a marked space at the opposite end of the tunnel.

Cognitive performance was tested using a Stroop Colour and Word test [60, 61]. Each trial lasted for two minutes during which words were presented one at a time on the computer screen, each word with two alternative answers. The participant answered the alternative they thought to be correct by clicking on the right or left mouse button. The task was to correctly determine the font colour of the written word as fast as possible, apart from the case when the font colour was red, in which case the correct answer was the written word. The number of correct answers in relation to the number of words completed (% correct answers), as well as the absolute value for number of completed words during each test, was used for statistical analysis. 

*Metabolic Expenditure*. Metabolic expenditure was measured in kilocalories (kcal) using data provided by the office-cycle which was extracted using Monark 939E Analysis Software and Microsoft Excel version 14.4.7. The office-cycle logged the effect (W) with a sample frequency of 1 Hz and the energy in Joule (W/fs) was converted to kcal using the constant 0,0002388 [62]. The metabolic expenditure (kcal) was then calculated using the algorithm (W/fs)*∗*0,0002388*∗*5. The constant of 5 was used according to standards applied by Monark Exercise AB and is an estimate based on the knowledge regarding the work efficiency (external power divided by total energy expenditure) of the human body's metabolism during cycling which is within the range of 10–25% [63]. Data regarding energy expenditure was also collected using a SenseWear® Armband (display model DD100, BodyMedia®), which was placed on the participants upper left arm. The data was extracted using SenseWear version 8.0. The SenseWear Armband uses data collected through a 3-axis accelerometer, heat flux sensor, galvanic skin response sensor, skin temperature sensor, and a near-body ambient temperature sensor along with demographic characteristics (age, gender, height, and weight) [64]. Absolute metabolic expenditure presented in kilocalories (kcal) as well as metabolic expenditure rates (kilocalories per minute (kcal/min)) was calculated for each individual and condition and used in the comparisons between the conditions.

### 2.4. Sample Size Determination

A power calculation based on a two-way analysis of variance with two factors, test condition and time (order of test conditions), using reference data from 10 healthy individuals on test-retest measures in PPT for the upper trapezius (mean difference 40.3 SD 32.4 kPa) resulted in a required sample of 36 participants to achieve balance between test sequences, a power > 0.8 with *α* = 0.5 and medium effect size (Cohens *d* 0.5).

### 2.5. Statistical Analysis

Demographic data and baseline characteristics are presented as mean and standard deviation (SD). PPT and TPT data are presented as group median and interquartile range (IQR) for the preactivity test occasion as well as median differences within the same experimental condition between the preactivity test occasion and the two postactivity test occasions. Descriptive data regarding work performance (mouse pointing task, transcription task, and Stroop Colour and Word test) is presented as a group median for each test occasion during each experimental condition as well as a group median based on all the individual mean values of the test results during the same experimental condition. Metabolic expenditure is presented as median and IQR of total metabolic expenditure as well as metabolic expenditure rate (kcal/min).

Differences in PPT measures and differences in TPT values were statistically analysed both within the same experimental condition and between the three experimental conditions. Differences in work performance between the three experimental conditions were statistically analysed using each individual mean value of the two test occasions (at 10 and 20 minutes of activity) for the condition.

All data were analysed using IBM SPSS Statistics 23.0. Statistical comparisons of the changes in the different outcome variables between the test occasions within the same condition were made using the nonparametric test Wilcoxon signed rank test. Comparisons of the outcome variables between the three different conditions were made either using Friedman's related samples two-way analysis of variance by ranks and post hoc tests by Wilcoxon signed rank test or using repeated measures ANOVA and post hoc* t*-tests when appropriate. Comparisons between the conditions regarding TPT results were made using Kruskal-Wallis one-way analysis of variance since floor and ceiling effects of the TPT test resulted in unrelated samples for this outcome measure. For all statistical tests a significance level of *α* < 0.05 was accepted.

## 3. Results 

Sixty-four individuals were interested in participating out of whom 41 individuals fitted the inclusion criteria and started the data collection. One participant dropped out due to relocation to another city and two due to health issues unrelated to the study. Another two participants were excluded from analysis due to incomplete data sets. In total 36 individuals completed the full data collection; baseline characteristics are presented in Table 2. The mean (SD) power output was 41.9 (10.6) and 104.3 (26.5) watts during LC and MC, respectively. Self-reported ratings of perceived exertion (Borg-RPE, 6–20) at the last minute of activity during each condition were 7.6, 11.2, and 14.7 during standing, LC, and MC respectively.

### 3.1. Pain Modulation

There were no significant differences between the preactivity values for any of the PPT test sites for any of the conditions (Table 3). Within the same condition there were significant increases in PPT for QF, VF, and TP both directly after and 30 minutes after completed activity as well as at 30 minutes of cycling compared to preactivity during LC (*p* values < 0.001–0.015). MC also resulted in significant increases in PPT both directly after and 30 minutes after completed activity (*p* values < 0.001–0.002). The control condition, standing, also showed significant increases in PPT both directly and 30 minutes after completed activity for QF and TP (*p* values < 0.001–0.017) whilst for VF the increase was only significant 30 minutes after completing the activity (*p* < 0.001). The only significant difference between the conditions was the change in PPT for trapezius where the difference directly after completing the activity compared to preactivity was 16.8, 39.3, and 17.0 kPa for standing, LC, and MC, respectively (*p* = 0.015). Post hoc analysis showed a larger increase in PPT for trapezius after LC compared to after standing (*p* = 0.014) but not when compared to after MC (*p* = 0.072), nor was there any difference in the increase in PPT for trapezius after MC compared to after standing (*p* = 0.362).

Individuals who reached minimum (5°C) or maximum (52°C) temperatures at the preactivity TPT test were excluded from that particular analysis (range of 1–15 participants excluded, see Table 4 for details). Median temperatures and differences in TPT directly after completed activity and 30 minutes after completed activity compared to preactivity are presented in Table 4. There were no significant differences within any of the conditions regarding CPT or HPT of the two test sites, nor were there any significant differences between the three conditions regarding changes in TPT either for CPT or HPT for any of the test sites.

### 3.2. Work Performance

Results from the three work performance tests are presented in Table 5. There were significantly more typing errors made during LC and MC compared to when standing (*p* = 0.009 and *p* < 0.001) but not when comparing LC to MC (*p* = 0.383). This resulted in faster gross typing speed during standing compared to both LC and MC (*p* = 0.017 and *p* = 0.009) although there was no difference in gross typing speed when comparing LC to MC (*p* = 0.333). The net typing speeds were significantly slower during the cycling conditions compared to during standing (*p* = 0.003 and *p* = 0.002 when compared to LC and MC, resp.) but not when comparing LC to MC.

In the mouse pointing task participants had less precision during both MC and LC when compared to standing (*p* < 0.001). Post hoc analysis also showed that there were a significantly lower number of successful trials during both LC and MC compared to standing as well as during MC compared to LC (all* p* values were <0.001). During LC the participants were significantly faster in completing the 10 mouse pointing trials compared to when standing (*p* = 0.002) but there was no difference in time it took to complete the trials between LC and MC (*p* = 0.962). During MC the participants were instead significantly slower in completing the trials compared to when standing (*p* = 0.045).

From the analysis obtained from the Stroop test, it was found that there were no significant differences between the conditions regarding either number of completed words or the percentage of correct answers.

### 3.3. Metabolic Expenditure

**Figure 1:**
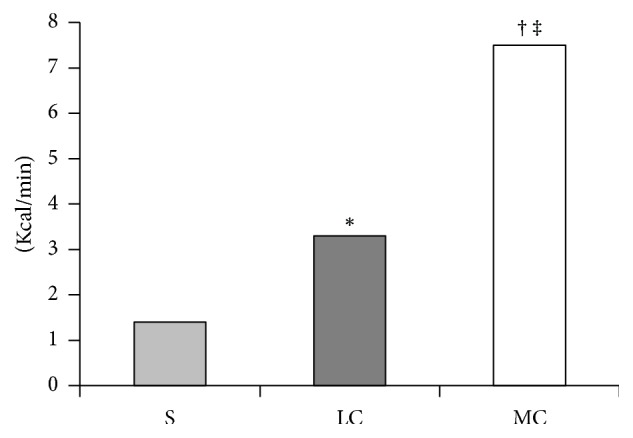
Metabolic expenditure rates were 1.4 (1.3; 1.7), 3.3 (2.3; 3.7), and 7.5 (5.8; 8.7) kcal/min during standing (S), low intensity cycling (LC), and moderate intensity cycling (MC), *p* < 0.001, with post hoc analysis showing significant differences in all three pairwise comparisons.^*∗*^LC versus S, ^†^LC versus MC, and ^‡^MC versus S, all *p* < 0.001. Kcal/min = kilocalories per minute.

Median (IQR) total energy expenditure was 43.0 (38.0; 50.8), 250.0 (173.9; 278.5), and 225.0 (173.8; 260.3) kcal during standing, LC, and MC, respectively, and differed significantly between all three conditions (*p* < 0.001). The energy expenditure rates also differed significantly between all three conditions (Figure 1).

## 4. Discussion

All conditions resulted in significantly increased PPTs, that is, the participants became less sensitive to pressure, on all test sites both directly after completing the activity and 30 minutes after completion, apart from VF directly after completing the 30 minutes of standing. The increase in PPT for trapezius was significantly higher directly after completing LC (39.3 kPa) when compared to MC and S (17.0 and 16.8 kPa). There were effects on work performance; typing performance (net and gross typing speed and number of typing errors) was impaired during MC but only slightly so during LC. Mouse pointing showed mixed results with reduced time used to complete the 10 trials during LC but with an impaired success rate during both cycling conditions. There were no significant differences between the conditions regarding the cognitive Stroop Colour and Word test. Metabolic expenditure rates differed significantly between all three conditions with median rates of 7.5, 3.3, and 1.4 kcal/min during MC, LC, and standing.

### 4.1. Pain Modulation

In this study, the PPT for all test sites increased significantly after standing and cycling on both intensities. There was however no difference in the amount of increase between the three conditions apart from the difference in pre- versus postactivity measurements for PPT trapezius where the largest increase was seen after LC. However, a minimal detectable change (MDC) of 42,7 kPa for upper trapezius has been suggested [65] and the changes seen in this study were only above this MDC value when comparing PPT measurements 30 min after activity with the case before activity during the LC and MC conditions. Even though not seen in this study, previous research has found indications of dose-response relationship between the intensity and duration of exercise and the effects on pain perception [66] and the largest effects on pressure pain thresholds were seen after high intensity exercise (75% of VO_2_max) with durations longer than 10 minutes. A more recent study confirmed this assumed dose-response relationship between the intensity of the exercise and effects on pain perception, presenting larger effects after exercising at vigorous intensity (20 minutes of cycling at 70% of heart rate reserve) than after moderate intensity exercise (20 minutes of cycling at 50–55% of heart rate reserve) [39].

### 4.2. Work Performance

There were small but significant impairments in work performance whilst cycling. The participants net and gross typing speed were significantly slower during both LC and MC when compared to standing. The difference in net speed between standing and LC was 2 words/min which may not result in any relevant productivity loss. Typing speed has been showed to slow down during cycling at both 40 W and 80 W when compared to sitting [35]. However, cycling on a self-selected intensity (mean 38 SD 14 W) does not seem to negatively affect the participants typing performance when compared to sitting [34]. Another study found no difference in typing performance when cycling at 30% of the individual maximal external power compared to when sitting at a desk chair [36]. In the aforementioned study the participants were able to familiarise themselves twice with all the computerized tests, once whilst sitting and once whilst cycling. This may indicate that typing performance whilst cycling improves with practice and can reach the same levels as when sitting.

The 10 mouse pointing trials were completed slightly, but significantly faster during LC and slower during MC compared with standing. Mouse pointing precision was significantly impaired during both LC and MC when compared to standing. The impairments seen in mouse pointing performance are somewhat in line with ones presented by Carr et al. [67]. They found significantly impaired mouse aiming ability during seated pedalling (with back support) at a very low intensity (9 W) compared to when using a seated sedentary workstation. They did not however find any significant differences in their mouse drag task nor menu navigation when comparing the results from the two different workstations. It might be that a cycling workstation is not a suitable option for increasing light intensity activity during the work day if the work involves a lot of precision mouse pointing tasks. This is notable since both low and very low intensity cycling have showed significant impairments in mouse pointing performance. However, no one has investigated the long term effects on mouse pointing performance and its possibility of improving with time as the user gets more and more accustomed to the active workstation.

Two studies evaluating work performance during treadmill walking and cycling both found that typing performance and mouse pointing performance were decreased to a greater extent when using a treadmill workstation compared to when cycling on an ergometer cycle [31, 68]. It was discussed that this might be due to a more stable base when seated compared to when walking, resulting in fewer upper body movements [69] and thus permitting the individual to focus better on the fine motor tasks of typing and mouse pointing. Our results showed that typing performance was hampered during MC and slightly so during LC when compared to standing still. This may be because cycling, as well as walking, is accompanied by uncontrolled upper body movements, which are not seen when standing still, when the upper body is not stabilized by holding on to handlebars. If habituation and/or practice improves this remains to be established in future studies.

Within the research field of associations between exercise and cognition, it has been confirmed that moderate intensity exercise has direct positive effects on processing speed with a moderate effect size [70] and a faster reaction time on cognitive control functions [71]. In the present study, we found no difference in cognitive performance between any of the conditions, which is in line with Torbeyns and coworkers comparing cycling (at 30% of maximal external power) and sitting [36]. The same study also found an improved response time in cognitive tests which is worth taking into consideration when discussing the use of active workstations and its effects on work performance. In another study, the participants used a cycling workstation at an exertion level comparable to normal walking pace and found that cycling did not affect complex cognitive performance but resulted in increased levels of positive affect, motivation, and morale compared to sitting [72].

Work productivity is of interest for not only the employer but also the employee in the context of using active workstations. A qualitative study recently presented perceived negative effects on work productivity as one of the barriers for implementing active workstations [73]. However, a review reported that studies that have evaluated acceptability of active workstations have predominantly reported positive feedback [74].

### 4.3. Metabolic Expenditure

In this present study metabolic expenditure rate (kcal/min) significantly differed between all three experimental conditions with median rates of 1.4, 3.3, and 7.5 kcal/min during standing, LC, and MC, respectively. The metabolic expenditure during standing was 1.4 kcal/min which is in line with previous studies showing metabolic expenditure rates of 1.29 and 1.36 kcal/min whilst standing [75, 76].

The fact that active workstations are an effective way of reducing sedentary behaviour has been highlighted in a recent meta-analysis which found a pooled effect size of reduced sedentary time of -77 minutes per 8-hour workday (95% CI = -120, -35 minutes) [74]. Another recent review summarizing the current knowledge regarding effects on metabolic expenditure when using active workstations also found that active workstations have shown promising results with mean energy expenditure rates ranging between 2 and 4 kcal/min compared to means ranging between 0.99 and 1.46 kcal/min when sitting [77].

There are however differences in metabolic expenditure rates depending on type of active workstation and, even more so, depending on given instructions to the participants. Some studies have given controlled instructions regarding the intensities, whilst others have let the participants use the active workstation on a self-selected intensity. The metabolic expenditure rate observed by Elmer and Martin [34], where 10 healthy participants cycled on a self-selected intensity (mean 51 SD 14 rpm and mean 38 SD 14 W), resulted in a mean rate of ≈4.17 kcal/min which is higher than the metabolic expenditure rate seen in this present study during LC (3.3 kcal/min) which corresponded to a fairly similar mean intensity (mean 41.9 SD 10.6 W). It is possible that the results from Elmer et al. are more reliable since they used circuit spirometry to measure metabolic expenditure whilst our study used data based on the work efficiency which is within the range of 10–25% [63], allowing for a larger error in the estimate.

Thus, in contrast to earlier studies that showed clear dose-response relationship between intensity of the exercise and effects on pain perception [39, 66], our study showed a similar increase in PPT for all test conditions with the exception of a greater increase in PPT for trapezius after LC compared to MC and standing. Our results revealed that typing performance was hampered during MC and slightly so during LC. An impairment in typing performance whilst cycling has both been confirmed [35] and refuted [34, 36] in previous studies. These discrepancies in results could partly be due to familiarisation of the task resulting in learning and also different intensities of cycling. The mouse pointing task was generally performed faster but with less precision whilst cycling compared to the case during standing in our study which is in line with results presented by Carr et al. [67]. However, the possibility of mouse pointing performance improving with long term practice remains to be investigated in future studies. Previous research has shown that exercise has a positive effect on processing speed [70] and a faster reaction time on cognitive control functions [71]. Conversely our study revealed no differences in cognitive performance between any of the conditions which confirm results previously presented by Torbeyns and coworkers [36]. Active workstations have previously shown good potential in increasing metabolic expenditure rates (means ranging between 2 and 4 kcal/min) when compared to sitting (means ranging between 0.99 and 1.46 kcal/min) [77]. This was confirmed by our results showing higher metabolic expenditure rates whilst cycling (median 3.3 and 7.5 kcal/min during LC and MC) compared to standing (median 1.4 kcal/min).

Thus, for the implication of the present study, it appears that long term low intensity cycling may be a feasible option for an active workstation. This must however be pursued with further studies on people with musculoskeletal disorders as well as field studies in real life office environments.

### 4.4. Study Limitations

Many participants were excluded from the TPT analysis (Table 4) since they reached minimum or maximum temperatures during the preactivity test occasion, meaning that they only could change their heat or cold pain threshold in one direction.

The use of a submaximal aerobic exercise test may have led to an over- or underestimation of the participants' aerobic capacity, resulting in participants working on an intensity that did not correspond to their true 20% or 50% of MAP. Using a submaximal test gives, however, a more random variation in the error of estimating the maximum oxygen uptake in contrast to a test of maximum, or peak, oxygen uptake during a graded exercise test. The latter could create a more systematic variation in the measurement error, assuming well trained individuals are more used to exercising at high intensities and therefore also more likely to be able to push themselves to their true VO_2_max, whilst less fit individuals are more likely to give in too early and therefore be underestimated, as was recently addressed by Poole and Jones [78].

One of the objectives was to explore if static cycling has any additional health related benefits compared to the more common, already available, workplace alternative; standing. A sitting condition was excluded from this study since typing performance [31] as well as energy expenditure [77] has been shown to be comparable when sitting and standing. EIH has previously been confirmed after exercising during 15 minutes at an intensity of 75% of VO_2_max in healthy, both active and inactive subjects [40], as well as in individuals with chronic MS pain [41]. However, 75% of VO_2_max is unlikely to be a plausible intensity to combine with office work and was therefore excluded from this study. An experimental condition of cycling at 50% of MAP was chosen since exercising during 25 minutes at 50% of heart rate reserve previously has shown effects on pain perception [39]. The results from this study, however, indicate that even cycling at 50% of MAP (104.3 W) is associated with negative effects on work performance and may not be suitable for an office work place. Also, a previous study showed that users are not willing to cycle at work on 80 W due to too much sweating, but this does not seem to be a problem when cycling on a lower intensity (40 W) [35]. Since active workstations mainly intend to increase light intensity activity a second cycling intensity of 20% of MAP was chosen.

## 5. Conclusion

In summary, our hypotheses were partially supported, showing that LC seems to be the preferred option since it raised PPTs, more than doubled metabolic expenditure, whilst minimally influencing work performance when compared to standing. These findings are interesting and require corroboration in field studies.

## Figures and Tables

**Table 1:** The protocol for the three experimental conditions. The outcome measures were taken either continuously during the experiment (metabolic expenditure) or at a specified time related to when the experimental activity started.

	Before start	10 min	20 min	30 min	60 min	65 min	75 min	105 min
LC	PPT TPT	Mouse pointing Typing performance Stroop Colour & Word RPE	Mouse pointing Typing performance Stroop Colour & Word RPE	RPE PPT (only trapezius)		Mouse pointing Typing performance Stroop Colour & Word RPE	RPE PPT TPT	PPT TPT

MC	PPT TPT	Mouse pointing Typing performance Stroop Colour & Word RPE	Mouse pointing Typing performance Stroop Colour & Word RPE	RPE PPT TPT	PPT TPT			

Standing	PPT TPT	Mouse pointing Typing performance Stroop Colour & Word RPE	Mouse pointing Typing performance Stroop Colour & Word RPE	RPE PPT TPT	PPT TPT			

PPT = pressure pain threshold, TPT = thermal pain thresholds (heat and cold), RPE = ratings of perceived exertion, LC = low intensity cycling, and MC = moderate intensity cycling.

**Table 2:** Baseline characteristics.

	All	Female	Male
Sex	36 (100%)	21 (58%)	15 (42%)
Age, years	26.8 (7.6)	25.6 (8.1)	28.5 (6.7)
Height, cm	172.3 (10.4)	166.9 (8.4)	179.9 (7.9)
Weight, kg	71.0 (14.3)	67.0 (14.2)	76.5 (12.9)
Estimated VO_2_max, l/min	3.0 (0.7)	2.7 (0.6)	3.4 (0.7)
Test value, ml O2/kg*∗*min	42.5 (10.1)	41.1 (10.3)	44.5 (9.9)
MAP output 100%, watt	209.4 (52.1)	189.0 (43.7)	238.0 (50.7)

MAP output = maximum aerobic power output; VO_2_max = maximum oxygen uptake.

**Table 3:** Median (IQR) pressure pain thresholds (kPa) preactivity during the different conditions as well as median (IQR) differences between the preactivity measurement and the two postactivity measurements in pressure pain thresholds.

	Standing	LC	MC	*p* value
*Quadriceps femoris*				
Before activity	382.0 (297.6; 488.6)	346.5 (327.6; 513.5)	372.3 (278.5; 510.8)	0.973
Difference after activity and before activity	47.5 (−1.1; 70.0)^*∗∗∗*^	36.3 (−14.0; 69.9)^*∗∗*^	53.0 (5.4; 106.1)^*∗∗∗*^	0.472
Difference 30 min after activity and before activity	42.3 (12.1; 70.4)^*∗∗∗*^	66.3 (14.4; 116.5)^*∗∗∗*^	69.0 (10.4; 113.9)^*∗∗∗*^	0.303
*Ventral forearm*				
Before activity	308.0 (238.4; 398.5)	301.5 (229.5; 392.3)	285.3 (209.0; 391.9)	0.459
Difference after activity and before activity	8.0 (−22.1; 47.0)	40.3 (−2.6; 67.4)^*∗∗∗*^	25.0 (−1.4; 60.0)^*∗∗*^	0.178
Difference 30 min after activity and before activity	17.0 (1.3; 66.4)^*∗∗∗*^	53.3 (14.6; 85.6)^*∗∗∗*^	54.8 (14.0; 98.8)^*∗∗∗*^	0.174
*Trapezius*				
Before activity	294.0 (256.0; 331.1)	303.3 (203.5; 403.1)	270.8 (231.0; 404.5)	0.895
Difference after activity and before activity	16.8 (−5.6; 39.4)^*∗*^	39.3 (15.6; 78.6)^*∗∗∗*^	17.0 (2.8; 49.9)^*∗∗∗*^	**0.015**
Difference 30 min after activity and before activity	32.8 (2.3; 69.4)^*∗∗∗*^	52.0 (26.8; 88.9)^*∗∗∗*^	47.8 (27.5; 87.0)^*∗∗∗*^	0.252
Difference for 30 min cycling and preactivity		36.3 (3.4; 64.1)^*∗∗∗*^		0.256

IQR = interquartile range, kPa = kilopascal, LC = low intensity cycling, and MC = moderate intensity cycling. *p* values for nonparametric comparisons between conditions. Significant differences between conditions are indicated in bold. Significant changes within the groups are marked with ^*∗*^(*p* < 0.05), ^*∗∗*^(*p* < 0.01), or ^*∗∗∗*^(*p* < 0.001).

**Table 4:** Median (IQR) thermal pain thresholds (TPT) for the test sites neck and thenar muscle as well as difference in TPT between the preactivity measurement and the two postactivity measurements. *p* values for nonparametric comparisons between conditions.

	Standing	LC	MC	*p* value
*Neck *				
Cold preactivity	9.5 (7.4; 18.3)	13.2 (8.3; 21.6)	16.1 (10.8; 21.4)	0.263
Included participants, *n* (excluded, *n*)	23(13)	21 (15)	22 (14)	
Cold difference, before-after	−0.7 (−2.2; 0.8)	−1.4 (−3.4; 0.0)	−1.5 (−3.8; 0.6)	0.609
Cold difference 30 min after activity and before activity	−0.5 (−2.9; 2.5)	−1.4 (−3.9; 2.7)	−2.1 (−6.6; 1.1)	0.415
*Neck*				
Heat before activity	48.0 (45.0; 50.5)	47.6 (45.5; 50.2)	47.6 (42.2; 50.9)	0.861
Included participants, *n* (excluded, *n*)	33 (3)	30 (6)	31 (5)	
Heat difference, before-after	0.15 (−0.8; 1.1)	0.8 (0.2; 1.8)	0.2 (−1.2; 1.4)	0.142
Heat difference 30 min after activity and before activity	0.9 (−0.3; 1.5)	0.7 (−0.5; 1.7)	0.8 (−0.5; 1.6)	0.931
*Thenar muscle*				
Cold before activity	13.6 (8.0; 18.7)	15.2 (9.6; 21.7)	13.3 (8.9; 19.5)	0.536
Included participants, *n* (excluded, *n*)	31 (5)	30 (6)	32 (4)	
Cold difference, before-after	−0.5 (−1.7; 0.7)	−1.0 (−3.3; 0.4)	−0.3 (−3.3; 1.5)	0.585
Cold difference 30 min after activity and before activity	−1.1 (−3.2; 0.7)	−1.5 (−3.7; 0.7)	−1.6 (−3.6; 1.1)	0.852
*Thenar muscle*				
Heat before activity	45.3 (42.4; 48.8)	45.8 (42.9; 49.3)	46.1 (42.0; 49.6)	0.912
Included participants, *n* (excluded, *n*)	34 (2)	35 (1)	35 (1)	
Heat difference, before-after	0.3 (−0.3; 1.1)	0.3 (−0.5; 1.5)	0.6 (−0.5; 1.5)	0.800
Heat difference 30 min after activity and before activity	0.5 (−0.4; 1.5)	0.4 (−0.4; 1.9)	0.9 (0.0; 1.6)	0.605

IQR = interquartile range, TPT = thermal pain threshold, LC = low intensity cycling, and MC = moderate intensity cycling.

**Table 5:** Median (IQR) results for the individual mean results from test trials one and two during each condition. *p* values are shown for comparisons between conditions as well as significant post hoc tests.

	S	LC	MC	*p* value between conditions	Significant post hoc tests
*Typing performance*					
Gross speed	47.0 (38.3; 53.0)	46.5 (39.8; 51.6)	45.5 (37.6; 51.9)	0.007^*∗*^	S-LC, S-MC
Net speed	46.3 (36.8; 51.9)	44.3 (38.3; 48.8)	43.8 (36.0; 50.6)	0.001^*∗*^	S-LC, S-MC
Errors	13.8 (7.5; 21.9)	16.3 (10.0; 28.8)	20.0 (12.5; 30.0)	0.001^*∗*^	S-LC, S-MC
*Mouse pointing task*					
Successful tasks	7 (5.5; 8.0)	5.5 (4.5; 6.5)	3.5 (2.5; 5.5)	0.000^*∗*^	S-LC, S-MC, LC-MC
Time	33.6 (26.6; 38.7)	32.6 (28.4; 43.0)	33.9 (29.4; 41.8)	0.025^*∗*^	S-LC, S-MC
*Stroop Colour and Word test *					
Number of words	60.3 (53.0; 64.0)	56.0 (52.0; 63.0)	57.8 (52.0; 63.9)	0.441	
% correct words	97.8 (95.0; 98.9)	97.1 (94.4; 99.1)	96.2 (93.2; 98.7)	0.066	

IQR = interquartile range, S = standing, LC = low intensity cycling, and MC = moderate intensity cycling; ^*∗*^*p* < 0.05.

## References

[1] WHO http://whqlibdoc.who.int/publications/2010/9789241599979_eng.pdf.

[2] Hu G., Tuomilehto J., Borodulin K., Jousilahti P. (2007). The joint associations of occupational, commuting, and leisure-time physical activity, and the Framingham risk score on the 10-year risk of coronary heart disease. *European Heart Journal*.

[3] Chau J. Y., van der Ploeg H. P., Merom D., Chey T., Bauman A. E. (2012). Cross-sectional associations between occupational and leisure-time sitting, physical activity and obesity in working adults. *Preventive Medicine*.

[4] Van Uffelen J. G. Z., Wong J., Chau J. Y. (2010). Occupational sitting and health risks: A systematic review. *American Journal of Preventive Medicine*.

[5] Boyle T., Fritschi L., Heyworth J., Bull F. (2011). Long-term sedentary work and the risk of subsite-specific colorectal cancer. *American Journal of Epidemiology*.

[6] Zhang M., Xie X., Lee A. H., Binns C. W. (2004). Sedentary behaviours and epithelial ovarian cancer risk. *Cancer Causes & Control*.

[7] Chau J. Y., Grunseit A. C., Chey T. (2013). Daily sitting time and all-cause mortality: A meta-analysis. *PLoS ONE*.

[8] Duvivier B. M. F. M., Schaper N. C., Bremers M. A. (2013). Minimal intensity physical activity (standing and walking) of longer duration improves insulin action and plasma lipids more than shorter periods of moderate to vigorous exercise (cycling) in sedentary subjects when energy expenditure is comparable. *PLoS ONE*.

[9] Ekelund U., Steene-Johannessen J., Brown W. J. (2016). Does physical activity attenuate, or even eliminate, the detrimental association of sitting time with mortality? A harmonised meta-analysis of data from more than 1 million men and women. *The Lancet*.

[10] Cimmino M. A., Ferrone C., Cutolo M. (2011). Epidemiology of chronic musculoskeletal pain. *Best Practice & Research Clinical Rheumatology*.

[11] Hallman D. M., Gupta N., Mathiassen S. E., Holtermann A. (2015). Association between objectively measured sitting time and neck–shoulder pain among blue-collar workers. *International Archives of Occupational and Environmental Health*.

[12] Cagnie B., Danneels L., Van Tiggelen D., De Loose V., Cambier D. (2007). Individual and work related risk factors for neck pain among office workers: A cross sectional study. *European Spine Journal*.

[13] Lidwall U. https://www.forsakringskassan.se/wps/wcm/connect/d7d4b78e-39fa-4c2f-bed9-ade979b5ff23/socialforsakringsrapport_2015_1.pdf?MOD=AJPERES.

[14] Parent-Thirion A., Fernández Macías E., Hurley J., Vermeylen G. (2007). Fourth European Working Conditions Survey., Luxembourg. *Office for Official Publications of the European Communities*.

[15] Schneider E., Irastorza X., Copsey S. (2010). *OSH in figures: Work-related musculoskeletal disorders in the EU - Facts and figures*.

[16] Martimo KP., Shiri R., Miranda H., Ketola R., Varonen H., Viikari-Juntura E. (2009). Self-reported productivity loss among workers with upper extremity disorders. *Scandinavian Journal of Work Environment & Health*.

[17] Church T. S., Thomas D. M., Tudor-Locke C. (2011). Trends over 5 decades in U.S. occupation-related physical activity and their associations with obesity. *PLoS ONE*.

[18] Proper K., van Mechelen W. (September 2007). *Effectiveness and economic impact of worksite interventions to promote physical activity and healthy diet. Background paper prepared for the WHO/WEF Joint Event on Preventing Noncommunicable Diseases in the Workplace (Dalian/China, September 2007)*.

[19] John D., Thompson D. L., Raynor H., Bielak K., Rider B., Bassett D. R. (2011). Treadmill workstations: A worksite physical activity intervention in overweight and obese office workers. *Journal of Physical Activity & Health*.

[20] Koepp G. A., Manohar C. U., McCrady-Spitzer S. K. (2013). Treadmill desks: A 1-year prospective trial. *Obesity*.

[21] Ben-Ner A., Hamann D. J., Koepp G., Manohar C. U., Levine J. (2014). Treadmill workstations: The effects of walking while working on physical activity and work performance. *PLoS ONE*.

[22] Thompson W. G., Foster R. C., Eide D. S., Levine J. A. (2008). Feasibility of a walking workstation to increase daily walking. *British Journal of Sports Medicine*.

[23] Thompson W. G., Koepp G. A., Levine J. A. (2014). Increasing physician activity with treadmill desks. *Work*.

[24] Botter J., Burford E.-M., Commissaris D., Könemann R., Mastrigt S. H.-V., Ellegast R. P. (2013). The biomechanical and physiological effect of two dynamic workstations. *Lecture Notes in Computer Science (including subseries Lecture Notes in Artificial Intelligence and Lecture Notes in Bioinformatics): Preface*.

[25] Cox R. H., Guth J., Siekemeyer L., Kellems B., Brehm S. B., Ohlinger C. M. (2011). Metabolic cost and speech quality while using an active workstation. *Journal of Physical Activity & Health*.

[26] Levine J. A., Miller J. M. (2007). The energy expenditure of using a “walk-and-work” desk for office workers with obesity. *British Journal of Sports Medicine*.

[27] McAlpine D. A., Manohar C. U., McCrady S. K., Hensrud D., Levine J. A. (2007). An office-place stepping device to promote workplace physical activity. *British Journal of Sports Medicine*.

[28] Thompson W. G., Levine J. A. (2011). Productivity of transcriptionists using a treadmill desk. *Work*.

[29] Funk R. E., Taylor M. L., Creekmur C. C., Ohlinger C. M., Cox R. H., Berg W. P. (2012). Effect of walking speed on typing performance using an active workstation. *Perceptual and Motor Skills*.

[30] John D., Bassett D., Thompson D., Fairbrother J., Baldwin D. (2009). Effect of using a treadmill workstation on performance of simulated office work tasks. *Journal of Physical Activity & Health*.

[31] Straker L., Levine J., Campbell A. (2009). The effects of walking and cycling computer workstations on keyboard and mouse performance. *Human Factors: The Journal of the Human Factors and Ergonomics Society*.

[32] Carr L. J., Walaska K. A., Marcus B. H. (2012). Feasibility of a portable pedal exercise machine for reducing sedentary time in the workplace. *British Journal of Sports Medicine*.

[33] Parry S., Straker L., Gilson N. D., Smith A. J. (2013). Participatory workplace interventions can reduce sedentary time for office workers - A randomised controlled trial. *PLoS ONE*.

[34] Elmer S. J., Martin J. C. (2014). A cycling workstation to facilitate physical activity in office settings. *Applied Ergonomics*.

[35] Koren K., Pišot R., Šimunič B. (2016). Active workstation allows office workers to work efficiently while sitting and exercising moderately. *Applied Ergonomics*.

[36] Torbeyns T., De Geus B., Bailey S. (2016). Cycling on a bike desk positively influences cognitive performance. *PLoS ONE*.

[37] Koltyn K. F., Garvin A. W., Gardiner R. L., Nelson T. F. (1996). Perception of pain following aerobic exercise. *Medicine and Science in Sports and Exercise*.

[38] Drury D. G., Greenwood K., Stuempfle K. J., Koltyn K. F. (2005). Changes in pain perception in women during and following an exhaustive incremental cycling exercise. *Journal of Sports Science & Medicine*.

[39] Naugle K. M., Naugle K. E., Fillingim R. B., Samuels B., Riley J. L. (2014). Intensity thresholds for aerobic exercise-induced hypoalgesia. *Medicine & Science in Sports & Exercise*.

[40] Vaegter H. B., Handberg G., Jørgensen M. N., Kinly A., Graven-Nielsen T. (2015). Aerobic Exercise and Cold Pressor Test Induce Hypoalgesia in Active and Inactive Men and Women. *Pain Medicine*.

[41] Vaegter H. B., Handberg G., Graven-Nielsen T. (2016). Hypoalgesia after Exercise and the Cold Pressor Test is Reduced in Chronic Musculoskeletal Pain Patients with High Pain Sensitivity. *The Clinical Journal of Pain*.

[42] Nijs J., Kosek E., Van Oosterwijck J., Meeus M. (2012). Dysfunctional endogenous analgesia during exercise in patients with chronic pain: to exercise or not to exercise?. *Pain Physician*.

[43] Jones B., Kenward M. G. (1989). *Design and Analysis of Cross-Over Trials*.

[44] Macsween A. (2001). The reliability and validity of the Astrand nomogram and linear extrapolation for deriving VO2max from submaximal exercise data. *The Journal of Sports Medicine and Physical Fitness*.

[45] Hoffman M. D., Shepanski M. A., Ruble S. B., Valic Z., Buckwalter J. B., Clifford P. S. (2004). Intensity and duration threshold for aerobic exercise-induced analgesia to pressure pain. *Archives of Physical Medicine and Rehabilitation*.

[46] Ferguson B. (2014). ACSM’s Guidelines for Exercise Testing and Prescription 9th Ed. 2014. *The Journal of the Canadian Chiropractic Association*.

[47] Borg G. A. (1982). Psychophysical bases of perceived exertion. *Medicine and Science in Sports and Exercise*.

[48] Åstrand P-O., Rodahl K., Dahl H. A., Stromme S. B. (2004). *Physiotherapy Theory and Practice*.

[49] Meeus M., Roussel N. A., Truijen S., Nijs J. (2010). Reduced pressure pain thresholds in response to exercise in chronic fatigue syndrome but not in chronic low back pain: an experimental study. *Journal of Rehabilitation Medicine*.

[50] Newcomb L. W., Koltyn K. F., Morgan W. P., Cook D. B. (2011). Influence of preferred versus prescribed exercise on pain in fibromyalgia. *Medicine & Science in Sports & Exercise*.

[51] Morgan W. P., Horstman D. H. (1978). Psychometric correlates of pain perception.. *Perceptual and Motor Skills*.

[52] Ylinen J., Nykänen M., Kautiainen H., Häkkinen A. (2007). Evaluation of repeatability of pressure algometry on the neck muscles for clinical use. *Manual Therapy*.

[53] Lee H. S. (2014). The effects of aerobic exercise and strengthening exercise on pain pressure thresholds. *Journal of Physical Therapy Science*.

[54] Persson A. L., Brogådh C., Sjölund B. H. (2004). Tender or not tender: test-retest repeatability of pressure pain thresholds in the trapezius and deltoid muscle of healthy women. *Journal of Rehabilitation Medicine*.

[55] Fischer A. A. (1987). Pressure algometry over normal muscles. Standard values, validity and reproducibility of pressure threshold. *PAIN*.

[56] Pöntinen P. J. (1998). Reliability, validity, reproducibility of algometry in diagnosis of active and latent tender spots and trigger points. *Journal of Musculoskeletal Pain*.

[57] Wang R., Cui L., Zhou W., Wang C., Zhang J., Wang K. (2014). Reliability study of thermal quantitative sensory testing in healthy Chinese. *Somatosensory & Motor Research*.

[58] Johnston V., Jimmieson N. L., Jull G., Souvlis T. (2008). Quantitative sensory measures distinguish office workers with varying levels of neck pain and disability. *PAIN*.

[59] Ruble S. B., Hoffman M. D., Shepanski M. A., Valic Z., Buckwalter J. B., Clifford P. S. (2005). Thermal pain perception after aerobic exercise. *Archives of Physical Medicine and Rehabilitation*.

[60] Alderman B. L., Olson R. L., Mattina D. M. (2014). Cognitive function during low-intensity walking: A test of the treadmill workstation. *Journal of Physical Activity & Health*.

[61] Ohlinger C. M., Horn T. S., Berg W. P., Cox R. H. (2011). The effect of active workstation use on measures of cognition, attention, and motor skill. *Journal of Physical Activity & Health*.

[62] Nordling C., Österman J. (2006). *Physics Handbook for Science and Engineering*.

[63] Gaesser G. A., Brooks G. A. (1975). Muscular efficiency during steady rate exercise: effects of speed and work rate. *Journal of Applied Physiology*.

[64] Wearable Multi Sensor Technology: Apex Medical NZ Ldt, http://apexmedical.co.nz/wp-content/uploads/2015/09/SensWear-Brochure-Sept-2015.pdf

[65] Walton D. M., Macdermid J. C., Nielson W., Teasell R., Chiasson M., Brown L. (2011). Reliability, standard error, and minimum detectable change of clinical pressure pain threshold testing in people with and without acute neck pain. *Journal of Orthopaedic and Sports Physical Therapy*.

[66] Naugle K. M., Fillingim R. B., Riley III J. L. (2012). A meta-analytic review of the hypoalgesic effects of exercise. *The Journal of Pain*.

[67] Carr L. J., Maeda H., Luther B., Rider P., Tucker S. J., Leonhard C. (2014). Acceptability and effects of a seated active workstation during sedentary work: A proof of concept study. *International Journal of Workplace Health Management*.

[68] Commissaris D. A. C. M., Könemann R., Hiemstra-van Mastrigt S. (2014). Effects of a standing and three dynamic workstations on computer task performance and cognitive function tests. *Applied Ergonomics*.

[69] Winter D. A. (1995). Human balance and posture control during standing and walking. *Gait & Posture*.

[70] McMorris T., Hale B. J. (2012). Differential effects of differing intensities of acute exercise on speed and accuracy of cognition: A meta-analytical investigation. *Brain and Cognition*.

[71] Davranche K., McMorris T. (2009). Specific effects of acute moderate exercise on cognitive control. *Brain and Cognition*.

[72] Pilcher J. J., Baker V. C. (2016). Task performance and meta-cognitive outcomes when using activity workstations and traditional desks. *Frontiers in Psychology*.

[73] Cifuentes M., Qin J., Fulmer S., Bello A. (2015). Facilitators and barriers to using treadmill workstations under real working conditions: A qualitative study in female office workers. *American Journal of Health Promotion*.

[74] Neuhaus M., Eakin E. G., Straker L. (2014). Reducing occupational sedentary time: A systematic review and meta-analysis of evidence on activity-permissive workstations. *Obesity Reviews*.

[75] Reiff C., Marlatt K., Dengel D. R. (2012). Difference in caloric expenditure in sitting versus standing desks. *Journal of Physical Activity & Health*.

[76] Speck R. M., Schmitz K. H. (2011). Energy expenditure comparison: A pilot study of standing instead of sitting at work for obesity prevention. *Preventive Medicine*.

[77] Tudor-Locke C., Schuna J. M., Frensham L. J., Proenca M. (2014). Changing the way we work: elevating energy expenditure with workstation alternatives. *International Journal of Obesity*.

[78] Poole D. C., Jones A. M. (2017). Measurement of the Maximum Oxygen Uptake ${\dot{V}o}_{2max}$: ${\dot{V}o}_{2peak}$ is no longer acceptable. *Journal of Applied Physiology*.

